# The Inhibition of Osteoblast Viability by Monosodium Urate Crystal-Stimulated Neutrophil-Derived Exosomes

**DOI:** 10.3389/fimmu.2022.809586

**Published:** 2022-05-17

**Authors:** Ertao Jia, Haiqiong Zhu, Hongling Geng, Li Zhong, Xia Qiu, Jingjing Xie, Yuya Xiao, Yubao Jiang, Min Xiao, Yanying Zhang, Jiaxin Wei, Dabin Tang, Jianyong Zhang

**Affiliations:** ^1^ The Department of Rheumatology, Shenzhen Traditional Chinese Medicine Hospital, Shenzhen, China; ^2^ The Department of Rheumatology, The Fourth Clinical Medical College of Guangzhou University of Chinese Medicine, Shenzhen, China; ^3^ Shenzhen Traditional Chinese Medicine Hospital Affiliated to Nanjing University of Chinese Medicine, Shenzhen, China; ^4^ The Department of Gynecology, Guangdong Provincial Hospital of Chinese Medicine, The Second Affiliated Hospital of Guangzhou University of Chinese Medicine, Guangzhou, China

**Keywords:** gout, neutrophil-derived exosomes, osteoclasts, osteoblasts, monosodium urate crystals

## Abstract

**Background and Objective:**

Bone erosion is common in patients with gout. The role of neutrophil-derived exosomes in gouty bone erosion remains elusive. This study aimed to investigate the functions of the neutrophil-derived exosomes in the development of bone erosion in gout.

**Methods:**

Neutrophil-derived exosomes were collected and assessed by transmission electron microscopy and nanoparticle tracking analysis. Cell counting kit-8 assay was applied to evaluate cell viability, and cell apoptosis was assessed by flow cytometry. In addition, quantitative Real-time PCR and Western blotting were used to determine the expression levels of alkaline phosphatase (ALP), osteoprotegerin (OPG), and receptor activator of nuclear factor-κB ligand (RANKL). Neutrophil-derived exosomes were tagged with PKH67. The miRNA expression profiles of exosomes and human fetal osteoblasts (hFOB) were compared using high-throughput sequencing. Functional miRNAs transfected into hFOB after co-incubation with exosomes were selected and validated by preliminary qPCR.

**Results:**

Neutrophil-derived exosomes were stimulated by monosodium urate (MSU). The exosomes could inhibit the viability of the hFOB, and the expression levels of ALP and OPG were down-regulated, while the expression level of RANKL was up-regulated. However, there was no significant difference in the viability of osteoclasts and the expression of nuclear factor of activated T cells 1. Exosomes were observed in the cytoplasm under a confocal microscopy, confirming that exosomes could be taken up by hFOB. In total, 2590 miRNAs were found, of which 47 miRNAs were differentially expressed. Among the delivered miRNAs, miR-1246 exhibited the highest level of differential expression. The viability of hFOB was reduced by miR-1246 mimics and increased by miR-1246 inhibitors. There was no significant difference in hFOB apoptosis rate between the miR-1246 mimic and miR-1246 inhibitor group. MiR-1246 overexpression decreased the expression levels of ALP and OPG, whereas increasing the expression level of RANKL. In contrast, miR-1246 inhibitor increased the expression levels of ALP and OPG, while decreasing the expression level of RANKL. Neutrophil-derived exosomes stimulated by MSU could increase the expression of miR-1246.

**Conclusion:**

Neutrophil-derived exosomes stimulated by MSU could inhibit the viability of osteoblasts.

## Introduction

Gout is the most common inflammatory arthropathy caused by the deposition of MSU. The overall prevalence of gout was 3.9% in the United States (1). In patients with advanced gout, bone erosions are frequently detected by radiography. Catabolic changes such as bone erosion and cartilage loss, as well as anabolic new bone formation (NBF), occur in affected joints (2). Imaging-based studies have confirmed that tophus infiltration into bone is the dominant mechanism underlying the development of bone erosion (3, 4).

The pathogenesis of joint damage has still remained elusive. A magnetic resonance imaging (MRI)-based study demonstrated that cartilage damage was not associated with bone marrow edema (BME) in patients with gout (5), suggesting that inflammation did not play a role in the pathophysiology of joint damage. Osteocytes are important cells regulating bone remodeling. In addition, bone erosion in gout occurs at the tophus-bone interface by altering physiological bone turnover, which is associated with excessive osteoclastogenesis and reduced osteoblastic differentiation of bone marrow-derived mesenchymal stem cells (BMSCs). MSU affects the viability of osteocytes and indirectly promotes the transformation of osteocyte function to a pro-inflammatory and pro-resorptive state (6). The viability of osteoblasts is the rate-limiting step in bone remodeling, and the number and function of osteoblasts determine changes in bone mass (7). In addition to stimulating an increase in osteoclast number and bone resorption, pro-inflammatory cytokines are closely related to the development and function of osteoblasts. MSU, arising as a result of persistent hyperuricemia, induces matrix metalloprotease (MMP) expression in chondrocytes (8).

Neutrophils stimulated with MSU show a significantly delayed apoptosis (9, 10). Neutrophil-derived exosomes can enhance the release of anti-inflammatory mediators, exacerbating the inflammatory responses (11, 12). Extracellular vesicles (EVs) are submicron-sized lipid-bilayer enclosed vesicles released by cells (13). A variety of molecules including proteins, DNA fragments, RNAs, lipids, and metabolites can be selectively encapsulated into EVs and delivered to nearby and distant recipient cells (14). Exosomes are released from different types of healthy or tumor cells, and has been previously confirmed to be involved in cancer development through modulating metastasis, angiogenesis, and epithelial−to−mesenchymal transition (EMT) (15). The present study aimed to assess the functions of the neutrophil-derived exosomes in the development of bone erosion in patients with gout.

## Materials and Methods

### Materials

HL-60 (Cat. No. CC1903), hFOB1.19(Cat. No. CC4005) and THP-1cells (Cat. No. CC1904) were purchased from Cellcook (Guangzhou, China). Iscove’s modified Dulbecco’s medium (IMDM; Cat. No. C12440500 BT), DMEM/F12 (Cat. No. C11330500 BT) and Roswell Park Memorial Institute (RPMI)-1640 medium (Cat. No. C11875500 BT) were purchased from Gibco (New York, NY, USA). In addition, 2.5% trypsin (10X; Cat No. 15090046) was purchased from Gibco. Cell counting kit-8 solution (Cat. No. C0040) was purchased from Beyotime (Shanghai, China). APC Annexin V (Cat. No. KGA1022) was provided by KeyGEN BioTECH Co., Ltd. (Nanjing, China). Besides, 7-AAD (00-6993-50) was purchased from eBioscience Inc. (San Diego, CA, USA). M-MLV Reverse Transcriptase (Cat. No. M1705) and GoTaq^®^ qPCR Master Mix (Cat. No. A6002) were obtained from Promega (Madison, WI, USA). The main instruments used in this study were as follows: Microfuge 20R refrigerated centrifuge (Beckman Coulter Inc., Brea, CA, USA), ultra-low temperature refrigerator (Thermo Fisher Scientific, Waltham, MA, USA), Optima L-100XP ultracentrifuge (Beckman Coulter Inc.), transmission electron microscope (HT-7700; Hitachi, Tokyo, Japan), Flow NanoAnalyzer (NanoFCM, Beijing, China), incubator (Thermo Fisher Scientific), flow cytometer (Beckman Coulter Inc.), ultraviolet transmittance analyzer (Cat. No. UV-3A; HEMA Corp., Beijing, China), gene amplification instrument (Cat. No. Hema-9600; HEMA Corp.), 7900HT Fast Real-Time PCR System (Applied Biosystems, Waltham, MA, USA), and UP-250 ultrasonic homogenizer (Ningbo SCIENTZ Biotechnology Co., Ltd., Ningbo, China).

### Cell Culture

HL-60 cells (1 × 10^5^ cells/ml) were cultured in a RPMI-1640 medium supplemented with 1.3% DMSO in a humidified atmosphere containing 5% CO_2_ at 37°C for 3 days (16). hFOB were cultured in DMEM/F12 medium. THP-1 cells were cultured in a RPMI-1640 medium modified with 10% FBS, 0.05mM β-mercaptoethanol and 1% penicillin/streptomycin. THP-1 cells were then stimulated with 100ng/ml phorbol 12-myristate 13-acetate (PMA) for 48h. The supernatants containing neutrophil-derived exosomes were subsequently added to the THP-1 cell culture. The culture medium was changed every 3 days, and the cells were cultured with RANKL (50 ng/ml) and macrophage colony-stimulating factor (M-CSF, 25 ng/ml) for 7 days (17). The cell experiment was performed in triplicates.

### Characterization of Exosomes

#### Isolation of Exosomes

Neutrophils were induced from HL-60 cells (5×10^7^cells/ml), which were divided into two groups (negative control (NC) and MSU groups). The neutrophils were treated with MSU in the MSU group, and exosomes were isolated from neutrophils by ultracentrifugation. The supernatants were centrifuged at 2,000 ×g for 30 min (4°C) to eliminate the contamination of cells and cell debris, and further centrifuged at 12,000 ×g for 45 min (4°C). Then, the supernatant was filtered with a 0.45 μm syringe filter (Millipore, Burlington, MA, USA) and ultra-centrifuged at 110,000 ×g for 30 min (4°C) (Optima L-100XP; Beckman Coulter Inc.) to sediment the exosomes. After that, the exosomes were resuspended in PBS and precipitated again by ultracentrifugation at 110,000 ×g for 70 min (4°C).

#### Transmission Electron Microscopy (TEM)

A total of 5 ul exosomes obtained by ultracentrifugation were diluted and analyzed. Next, 10 μl phosphotungstic acid was dropped on a copper net, precipitated for 1 min, followed by floating liquid absorption with a filter paper at room temperature, drying at room temperature for 2 min, and was subjected to TEM.

#### Nanoparticle Tracking Analysis (NTA)

The size of the exosomes was analyzed using a Flow NanoAnalyzer (NanoFCM) equipped with nanoparticle particle tracking software. According to the manufacturer’s recommendation, the samples were illuminated with the laser, and the movement of nanoparticles due to Brownian motion was recorded for 60 s at a mean frame rate of 20 frames/s.

#### The Marker Proteins of Exosomes

The marker proteins of exosomes were examined using western blot. The primary antibodies, including anti‐CD9 (ab92726), anti‐TSG101 (ab125011) and anti‐Calnexin (ab22595), were purchased from Abcam. The goat anti-rabbit IgG peroxidase conjugated (AP132P) secondary antibody was obtained from Merck Millipore. Protein concentration was determined using a bicinchoninic acid (BCA) kit (BL521A), which was provided by Biosharp.

### Exosomes and Osteoblasts

#### Cell Proliferation Assay

Cell viability was examined using the CCK-8 assay. The cells in logarithmic growth phase were washed once with PBS, followed by digestion into a single cell suspension with 0.25% trypsin. Digestion was terminated by adding the complete medium and the cells were resuspended. The density of cells was adjusted to 3×10^4^ cells/ml. Next, 100 μL of culture medium was added into each well and incubated overnight in an incubator (37°C, 5% CO_2_). After cultivation for 0, 24, 48, and 72 h, CCK-8 reagent (dilution, 1:10) was added and the cells were incubated at 4 h for 30 min at 37°C. A microplate reader was used to detect the absorbance at a wavelength of 450 nm.

#### Flow Cytometry

Apoptosis was detected *via* flow cytometry. Briefly, cells (1.0×10^6^cells/ml) were washed in cold PBS. The washed cells were re-centrifuged, discarding the supernatant, and resuspended in 1X Annexin-binding buffer. Afterwards, 25 μL of the Annexin V conjugate and 2 μL of the 100 μg/mL propidium iodide (PI) working solution were added to each 100 μL of cell suspension. The cells were incubated at room temperature for 15 min and then were washed with 1X Annexin-Binding buffer.

#### Quantitative Reverse Transcription-Polymerase Chain Reaction

Herein, qPCR analysis was performed to determine the levels of alkaline phosphatase (ALP), receptor activator of nuclear factor-κB ligand (RANKL), osteoprotegerin (OPG), nuclear factor of activated T cells 1(NFATC1) and miRNA. Forward and reverse primer sequences can be found in **Table 1**. Total RNA was extracted from collected cells using the TRIzol reagent that was pre-cooled at 4°C. The cells were treated and the supernatant was collected. Next, chloroform (200 uL) was added to the supernatant. The supernatant was transferred into a new microcentrifuge tube (liquid phase without glass beads) pre-filled with 400 μl of the lower (liquid) phase with phenol/chloroform/isoamylalcohol. The suspension was mixed well for ~5 min by vortexing and centrifuged at 4°C at maximum speed for 10 min. The upper (aqueous) phase was collected in a new microcentrifuge tube and the same volume of cold isopropanol was added. The suspension was quickly vortexed and centrifuged at 4°C at maximum speed for 10 min. The liquid phase was carefully extracted and appropriately discarded, and the pellet was washed with 500 μl 3 M Na-acetate pH 5.2 (a short vortexing to help resuspend the pellet), followed by centrifugation at 4°C at maximum speed for 5 min. The pellet was then washed with 70% EtOH (short vortexing to help resuspend the pellet) and centrifuged at 4°C at maximum speed for 5 min. At the end of the washing step, the pellet was dried at room temperature in a laminar flow hood, resuspended in 30 μl DEPC-treated water at 60°C, and left in a 60°C incubator for 10 min. RNA (1000 ng) was treated with DNase I and reverse-transcribed to cDNA. RNA purities before and after DNase I treatment were measured spectrophotometrically (NanoDrop ND-1000 spectrophotometer, NanoDrop Technologies, Wilmington, DE, USA).

**Table 1:** The sequences of the primers.

Name	Sequences
ALP-R	GCAGTGAAGGGCTTCTTGTC
ALP-F	GGACATGCAGTACGAGCTGA
OPG-R	GAAGGTGAGGTTAGCATGTCC
OPG-F	CAAAGTAAACGCAGAGAGTGTAGA
RANKL-R	GGAACCAGATGGGATGTCGG
RANKL-F	TCGATGGCTCATGGTTAGATC
NFATC1-R	ACTTTGGTGTTGGAGAGGATGG
NFATC1-F	TGCTGCAGCTTTTCATTGGG
hsa-miR-3182-RT	GTCGTATCCAGTGCAGGGTCCGAGGTATTCGCACTGGATACGACGACTAC
hsa-miR-3182-F	GCTTCTGTAGTGTA
hsa-miR-6715b-3p-RT	GTCGTATCCAGTGCAGGGTCCGAGGTATTCGCACTGGATACGACCCACAG
hsa-miR-6715b-3p-F	CTCAAACCGGCTGTGCCT
hsa-miR-587-RT	GTCGTATCCAGTGCAGGGTCCGAGGTATTCGCACTGGATACGACGTGACT
hsa-miR-587-F	TTTCCATAGGTGATGAGT
hsa-miR-1246-RT	GTCGTATCCAGTGCAGGGTCCGAGGTATTCGCACTGGATACGACCCTGCT
hsa-miR-1246-F	CGCAATGGATTTTTGG
hsa-miR-335-5p	AACACGCTCAAGAGCAATAACG
hsa-miR-335-5p-RT	GTCGTATCCAGTGCAGGGTCCGAGGTATTCGCACTGGATACGACACATTT
hsa-miR-8055-RT	GTCGTATCCAGTGCAGGGTCCGAGGTATTCGCACTGGATACGACTCCGTC
hsa-miR-8055-F	CTTTGAGCACATGAGCAGA

#### Western Blot Analysis

Western blot analysis was performed to detect the expression levels of ALP, RANKL, and OPG. In brief, the cells were harvested by centrifugation, and then resuspended in PBS. Following sonication for cell lysis, the total protein concentration in the supernatants was measured using Bradford assay. Total protein (5 µg) was resolved by SDS-PAGE under non-reducing conditions, and subsequently transferred to a PVDF membrane in a Mini Trans-Blot Cell (Bio-Rad Laboratories Inc., Hercules, CA, USA) at 100 V for 2 h in Novex BoltTM transfer buffer (Thermo Fisher Scientific). After blocking with 5% skim milk in Tris-buffered saline with Tween 20 (TBS-T) for 1 h at room temperature, the PVDF membrane was incubated with mouse anti-FLAG antibody (1:5000; cat. no. F2555; Sigma-Aldrich; Merck KGaA) for 1 h at room temperature and sequentially probed with horseradish peroxide (HRP)-conjugated rabbit anti-mouse IgG antibody (1:5000; cat. no. A9044; Sigma-Aldrich). The secondary antibody was detected using a tetramethylbenzidine (TMB) substrate reagent according to the manufacturer’s instructions (BD Biosciences, San Jose, CA, USA). The chemiluminescent signals on the PVDF membrane were visualized using a ChemiDoc image system (Bio-Rad Laboratories Inc.).

#### Uptake of Exosomes

Neutrophil-derived exosomes were tagged with PKH67 green fluorescence cell linker mini kit (Sigma MINI67-1KT) in the light of the manufacturer’s instructions. Briefly, 1.5 mL EP was prepared for controlled exosome dilution. Exosomes were resuspended with 500 ul of Diluent C solution, and 2ul of PKH67 was added to 500ul Diluent C to prepare PKH67 Diluent. Next, the prepared exosome diluent and PKH67 diluent were mixed well and incubated at 37°C for 5min. Following incubation, 8 ml of 15% complete exosome-free medium was added to terminate the staining reaction. The exosomes were precipitated with an equal volume of PEG overnight at 4°C and centrifuged at 10,000 × g for 1 h at 4°C. The exosomes were resuspended in PBS (pH=7.4) and stored at −80°C. hFOB were loaded on the climbing slices one day earlier. On the second day, exosomes were added and incubated with cells for 24h, then washed with PBS for 3 times. Cells were fixed with 4% paraformaldehyde (PFA) for 10 min at room temperature, and permeabilized with 0.5% Triton X-100 in PBS for 5 min. After this, cells were then incubated with rhodamine phalloidin (Invitrogen R415) for 30 min. Nuclei were stained by adding DAPI (Beyotime C1005) for 10 min. Fluorescence was observed under a confocal microscopy.

### Exosomes and Osteoclasts

Osteoclast differentiation was induced with RANKL and MCSF after THP-1 cells were treated with PMA (100 ng/mL) for 24 h. Neutrophil-derived exosomes in the NC and MSU group were collected and co-incubated with osteoclast precursor cells induced by THP-1. Each fraction was denatured, and subjected to WB for identifying the osteoclast marker of NFATC1. Then, qPCR was used to detect the expression level of NFATC1. The primers of the related genes are listed in **Table 1**. This assay were carried out as described previously.

### Statistical Analysis

All data are presented as means ± standard deviation (SD). Comparisons were performed using t test and one-way ANOVA, as appropriate. Statistical analysis was performed using GraphPad Prism version 9.0. P≤ 0.05 was considered statistically significant.

## Results

### Characteristics of Neutrophil-Derived Exosomes Stimulated by MSU

**Figure 1:**
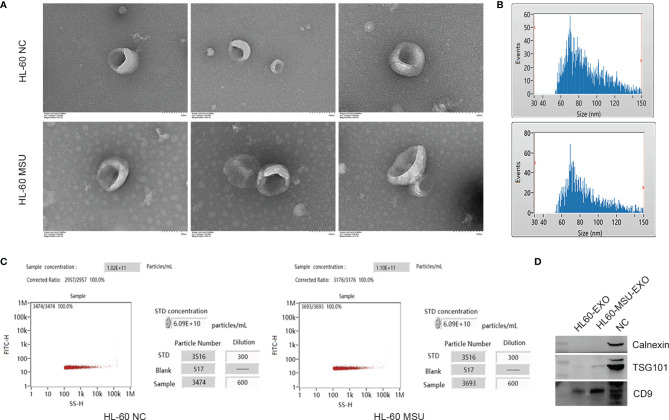
Characteristics of neutrophil-derived exosomes. **(A)** The size and shape of exosomes are observed by transmission electron microscopy (TEM). The size of exosomes ranges from 60 to 150 nm. **(B)** Schematically illustration of the distribution of exosome particle size. The particle size was assessed by nanoparticle tracking analysis (NTA). HL-60 NC: Median, 80.25 nm; Mean, 86.01 nm; SD, 21.05 nm; HL-60 MSU: Median, 82.25 nm; Mean, 87.69 nm; SD, 21.42 nm. **(C)** Schematically illustration of exosome particle concentration. **(D)** The exosome markers of CD9 and TSG101 are expressed obviously, and there is no expression of calnexin according to western blotting analysis.

Neutrophil-derived exosomes in the NC and MSU groups were extracted by ultracentrifugation. The morphology of exosomes that were uniform in shape and size was evaluated by TEM (**Figure 1A**). NTA was performed to analyze the distribution of particle size and density. The average particle size in the NC group was 86.01 nm, the proportion of particles with a size ranging from 30-150 nm was 95.1%, and the particle density was 1.02×10^11^ particles/mL. The average particle size in the MSU group was 87.69 nm, the proportion of particles with a size ranging from 30-150 nm was 95.47%, and the particle density was 1.10×10^11^ particles/mL (**Figures 1B, C**). The collected exosomes had a size range of 60-150 nm.

In order to confirm the isolated exosomes, western blotting was used to test the markers of exosome, including transmembrane protein (CD9), cytosolic proteins (TSG101) and endoplasmic reticulum protein (Calnexin). CD9 and TSG101 were expressed obviously, while there was no expression of calnexin (**Figure 1D**). The results indicated that there were abundant exosomes in the supernatant, which could be used for subsequent experiments.

### The Role of Neutrophil-Derived Exosomes on Osteoclasts and Osteoblasts

**Figure 2:**
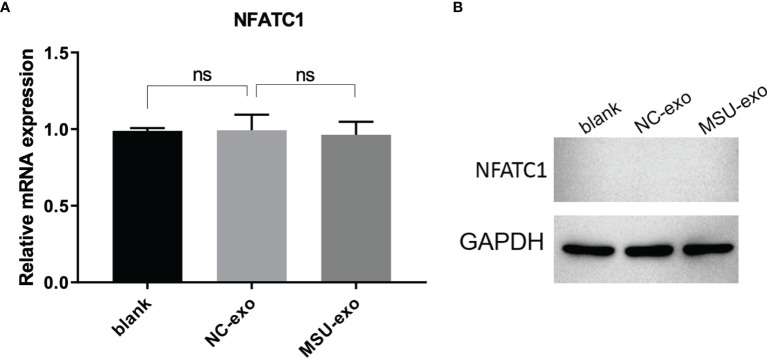
The effects of neutrophil-derived exosomes on osteoclasts. **(A)** QPCR is used to detect the expression of osteoclastic marker NFATC1, which did not significantly differ between the two groups. **(B)** The expression of osteoclastic marker NFATC1 is detected by western blot. There is no significant differences in the mRNA and protein level of NFATC1. ns, no significance.

To determine the role of neutrophil-derived exosomes on the viability of osteoclasts and osteoblasts, exosomes from the NC and MSU groups were co-incubated with THP-1 cells and hFOB for 48 h. No significant difference was found in the viability of osteoclasts. Besides, the expression level of nuclear factor of activated T cells 1 (NFATC1) did not significantly differ between the two groups (**Figure 2**).

**Figure 3:**
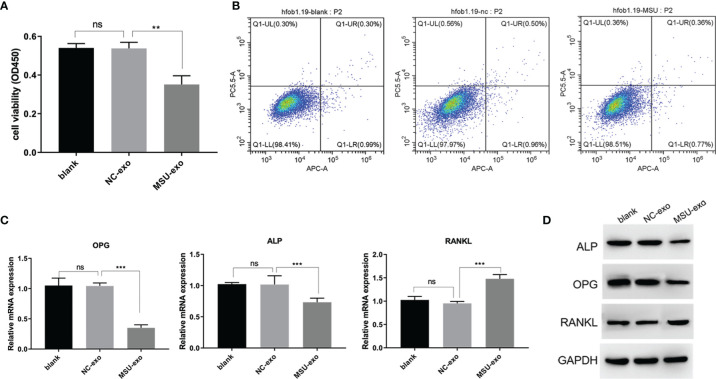
The effects of MSU-induced neutrophil-derived exosomes on hFOB. **(A)** CCK-8 assay is used to detect the cell viability. **(B)** Apoptosis is detected by flow cytometry. **(C)** QPCR was used to assess miRNA expression levels of ALP, OPG and RANKL. **(D)** Protein expression levels of ALP, OPG and RANKL in hFOB cultures were determined via western* *blot* *analysis. P<0.01**, P<0.001 ***determined by the t-test. ns, no significance.

Although the viability of hFOB did not significantly change in the NC group, that in the MSU group was inhibited (**Figure 3A**). The results of flow cytometry showed that there was no significant difference in the rate of apoptosis among the three groups (**Figure 3B**). The results of qPCR are illustrated in **Figure 3C**. It was found that there was no significant change in the levels of osteoblast markers between blank control and NC group. The miRNA levels of OPG and ALP in the MSU group were down-regulated, while the miRNA level of RANKL was up-regulated. It was noteworthy that western blotting and qPCR were used to simultaneously detect the expression levels of osteoblast markers. The protein levels of ALP, OPG, and RANKL are presented in **Figure 3D**. The results showed that there were no significant changes in the expressions of ALP, OPG, and RANKL between blank control and NC group, while the levels of OPG and ALP were down-regulated and the level of RANKL was up-regulated in the MSU group.

### Detection of Exosomes in Osteoblasts

**Figure 4:**
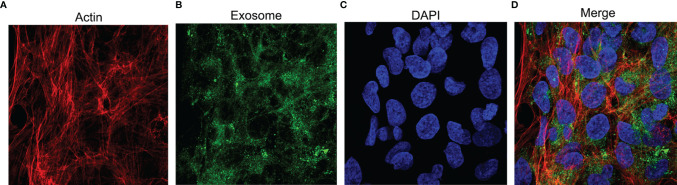
The uptake of exosomes by hFOB. The cells are co-incubated with exosomes for 24h and subjected to immunofluorescence staining. **(A)** TRITC Phalloidin labels the actin in red. **(B)** PKH67 labels exosomes in green. **(C)** The nuclei stained by DAPI appears blue. **(D)** Merge images.

Neutrophil-derived exosomes were co-incubated with hFOB. Rhodamine-phalloidin was used to stain the actin cytoskeleton (**Figure 4A**), the exosomes were fluorescently labelled using the PKH67 dye (**Figure 4B**) and DAPI was used to counterstain the nucleus (**Figure 4C**) as previously described. The fluorescence signal indicating uptake of exosomes was observed, photographed and merged (**Figure 4D**) using a confocal laser microscope system. The results represented the endocytosis of exosomes by osteoblasts.

### Exosomes Induced Differential Expression of miRNA in Osteoblasts

**Figure 5:**
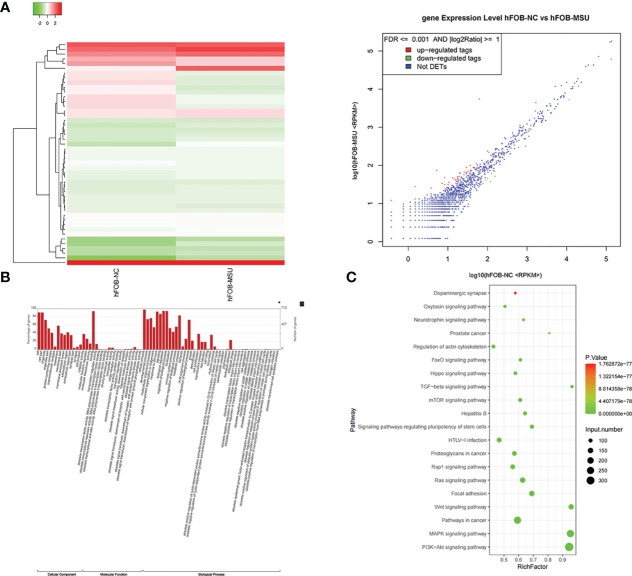
Exosomes induce differential expression of miRNA in hFOB. **(A)** Changes in miRNA expression in hFOB are detected. High-throughput sequencing is performed, and analysis of miRNA-seq data is conducted in both groups. **(B)** The target genes of differentially expressed miRNAs are predicted by Gene Ontology (GO) analyses. **(C)** KEGG analysis of target genes is performed. miRNA is enriched in many typical osteogenic pathways, including TGF-β, MAPK, PI-3K and Wnt pathway.

**Figure 6:**
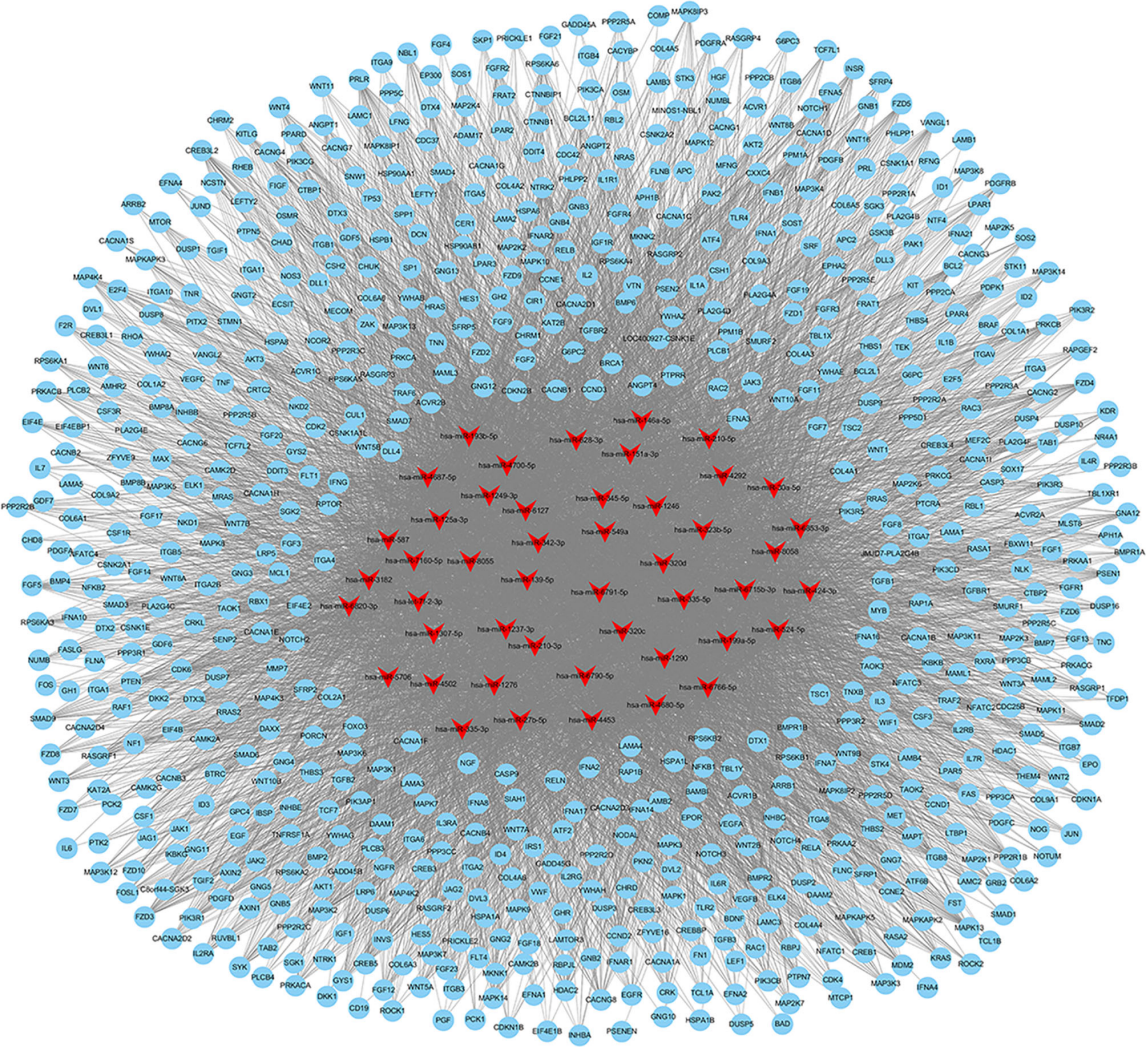
Networks of 717 enriched pathways associated with 47 differentially expressed miRNAs.

**Figure 7:**
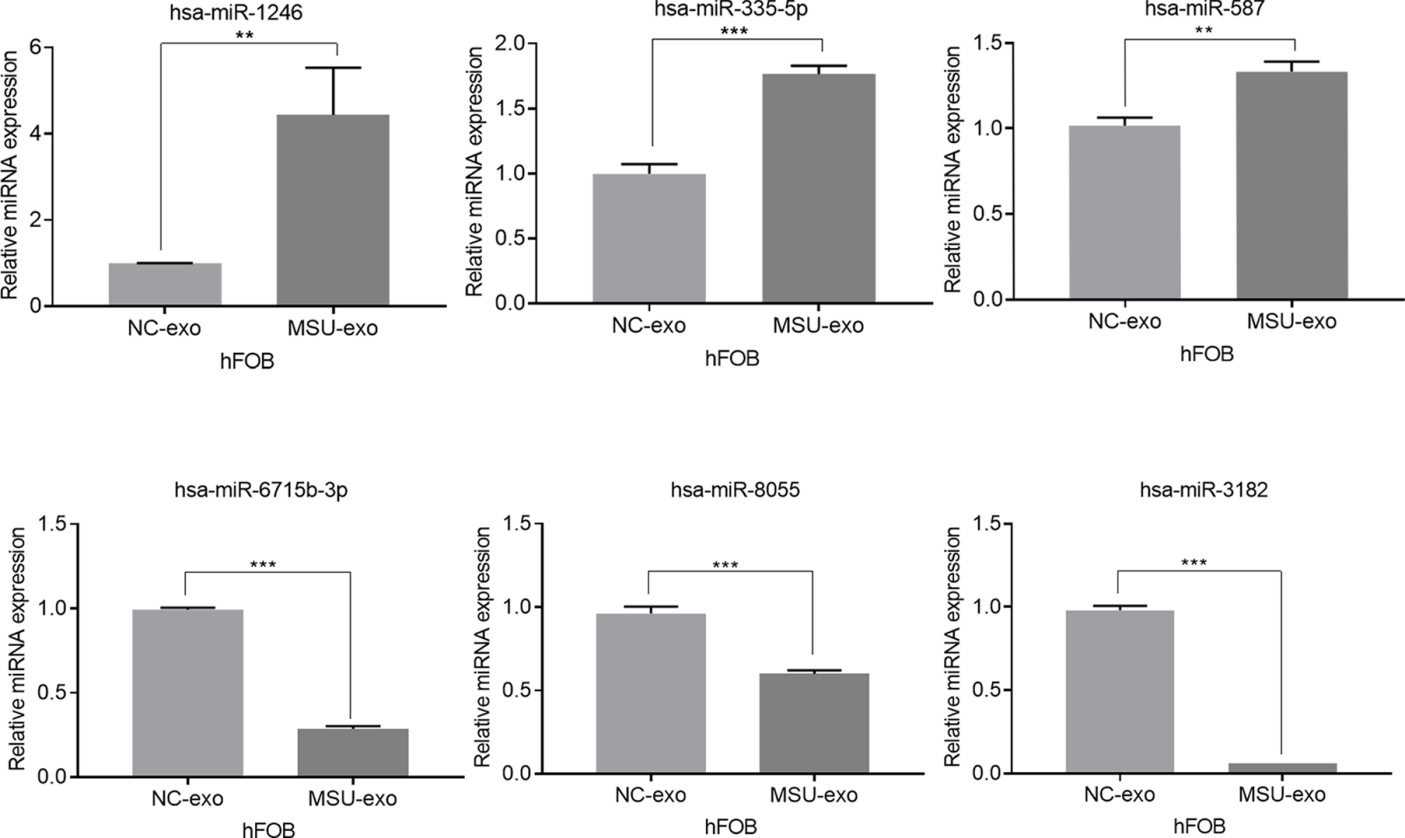
The three miRNAs with the highest and lowest expression levels are verified (**P < 0.01, and ***P < 0.001).

Exosomes in the supernatant of neutrophil culture in the NC and MSU groups were collected and co-incubated with hFOB. Changes in miRNA expression in hFOB were detected. In total, 2590 hFOB miRNAs were found in the two groups, of which 47 miRNAs were differentially expressed (log2 >1, false discovery rate (FDR)<0.001), including 35 up-regulated miRNAs and 12 down-regulated miRNAs in the MSU group (**Figure 5A**). Furthermore, Gene Ontology (GO) and Kyoto Encyclopedia of Genes and Genomes (KEGG) pathway enrichment analyses of differentially expressed genes were performed. The results of GO enrichment analysis were classified into three main sections, including cellular component (CC), molecular function (MF), and biological process (BP) (**Figure 5B**). The detailed results of KEGG pathway analyses are summarized in **Figure 5C**. Based on the KEGG pathway analyses, the 47 differentially expressed miRNAs were enriched in 717 pathways, such as TGF-β, MAPK, PI-3K pathway and other classical osteogenic pathways (**Figures 5C**, **6**). The three miRNAs with the highest and lowest expression levels were verified, which met the expectations (**Figure 7**).

### MiR-1246 Inhibited the Viability of Osteoblasts

**Figure 8:**
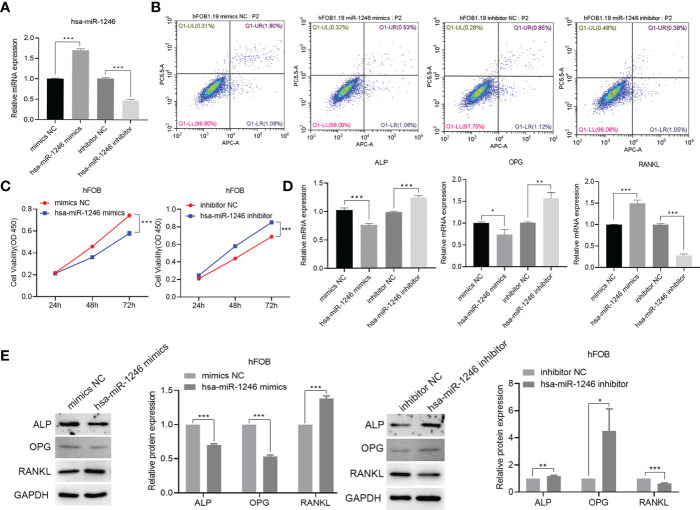
Construction of hsa-miR-1246 mimics and inhibitors. hFOB are cultured and transfected with hFOB-mimics NC, hFOB-inhibitor NC, hFOB-hsa-miR-1246 mimics, and hFOB-hsa-miR-1246 inhibitor. **(A)** Verified overexpression and interfering fragments by qPCR. **(B)** Cell apoptosis evaluated by flow cytometry. **(C)** Cell viability assessed using CCK-8. **(D)** QPCR to detect the expression levels of ALP, OPG, and RANKL. **(E)** Western blot analysis to determine the expression levels of ALP, OPG, and RANKL. (*P < 0.05, **P < 0.01, and ***P < 0.001).

We investigated the precise role of miR-1246 in hFOB, which was the most abundant miRNA. We transfected hFOB with miRNA mimics or inhibitors (**Figure 8A**). There was no significant difference in the apoptosis rate of hFOB between the two groups (**Figure 8B**). The results of CCK-8 assay showed that the viability of hFOB decreased by adding the miRNA mimics and increased by adding the miRNA inhibitors (**Figure 8C**). After the hFOB were transfected with miR-1246 mimics for 48 h, the levels of ALP and OPG were decreased, while the level of RANKL was increased. Transfection of miR-1246 inhibitors for 48 h resulted in an increase in the levels of ALP and OPG, and a decrease in the level of RANKL (**Figures 8D, E**).

**Figure 9:**
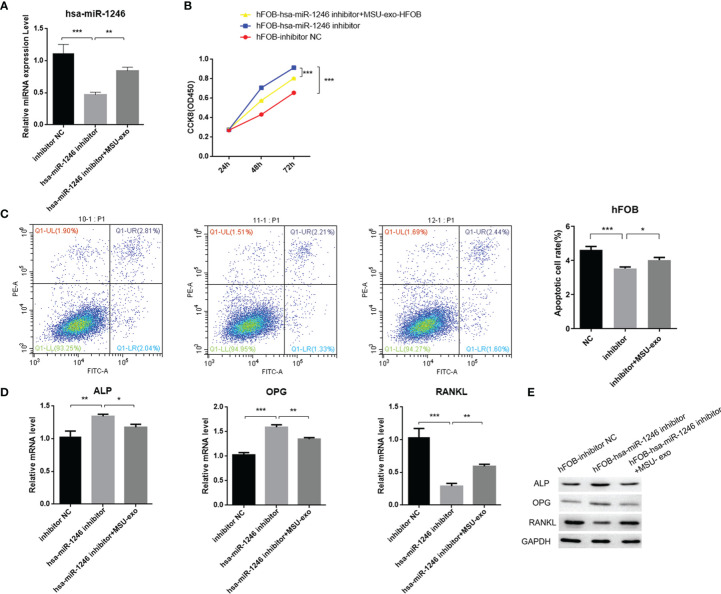
The effect of neutrophil-derived exosomes stimulated by MSU on viability of hFOB **(A)** Transfection of hFOB with miRNA inhibitors. Interfering fragments are verified by qPCR. **(B)** CCK8 assay to exam the cell viability. **(C)** Flow cytometric analyses to detect the percentages of apoptotic cells. **(D)** QPCR to detect the expression levels of ALP, OPG, and RANKL. **(E)** Western blot analysis to determine the expression levels of ALP, OPG, and RANKL (P < 0.05 *, P < 0.01 **, P < 0.001 ***).

In addition, to further explore the role of neutrophil-derived exosomes stimulated by MSU on the viability of hFOB, exosomes (1×10^8^ cells/ml) were co-incubated with hFOB transfected with hsa-miR-1246 inhibitor for 24 h. The results showed that exosomes could increase the expression level of miR-1246 (**Figure 9A**). CCK-8 assay suggested that proliferation and differentiation of osteoblast was promoted in the hFOB-hsa-miR-1246 inhibitor+MSU-exo group (**Figure 9B**), and the apoptosis rate of hFOB was increased compared with the miR-1246 inhibitor-transfection group (**Figure 9C**). The exosomes inhibited the expressions of ALP and OPG and increased the expression of RANKL (**Figures 9D, E**). These results indicated that miR-1246 could inhibit the viability of osteoblasts.

## Discussion

Bone erosion in gout is closely related to osteoblasts and osteoclasts. Under physiological conditions, RANKL regulates the differentiation of osteoclasts, and RANK is expressed in osteoclast precursors and can bind to the RANKL receptor on mature osteoblasts (18). A previous study found that the expression level of RANKL was elevated in gout patients, while the level of OPG was significantly reduced (19). However, the differentiation of osteoclasts could not be directly induced by MSU (19, 20). Another study reported that RANKL and OPG were independent factors for joint destruction in patients with gout (21).

Although MSU can directly inhibit the viability and function of osteoblasts (6, 22), accumulated MSU and other immune cells form a granuloma around the tophi with extensive bone resorption around them (23). Tophi can induce abundant neutrophils (24). Neutrophils play a critical role in gout. Neutrophils stimulated by MSU could promote the influx of neutrophils through several mechanisms, and the cell could release a series of substances (25).It was demonstrated that osteoblasts exposed to MSU could promote neutrophils to adhere to osteoblasts (8). The functions of neutrophils in bone erosion in patients with gout are still mysterious.

**Figure 10:**
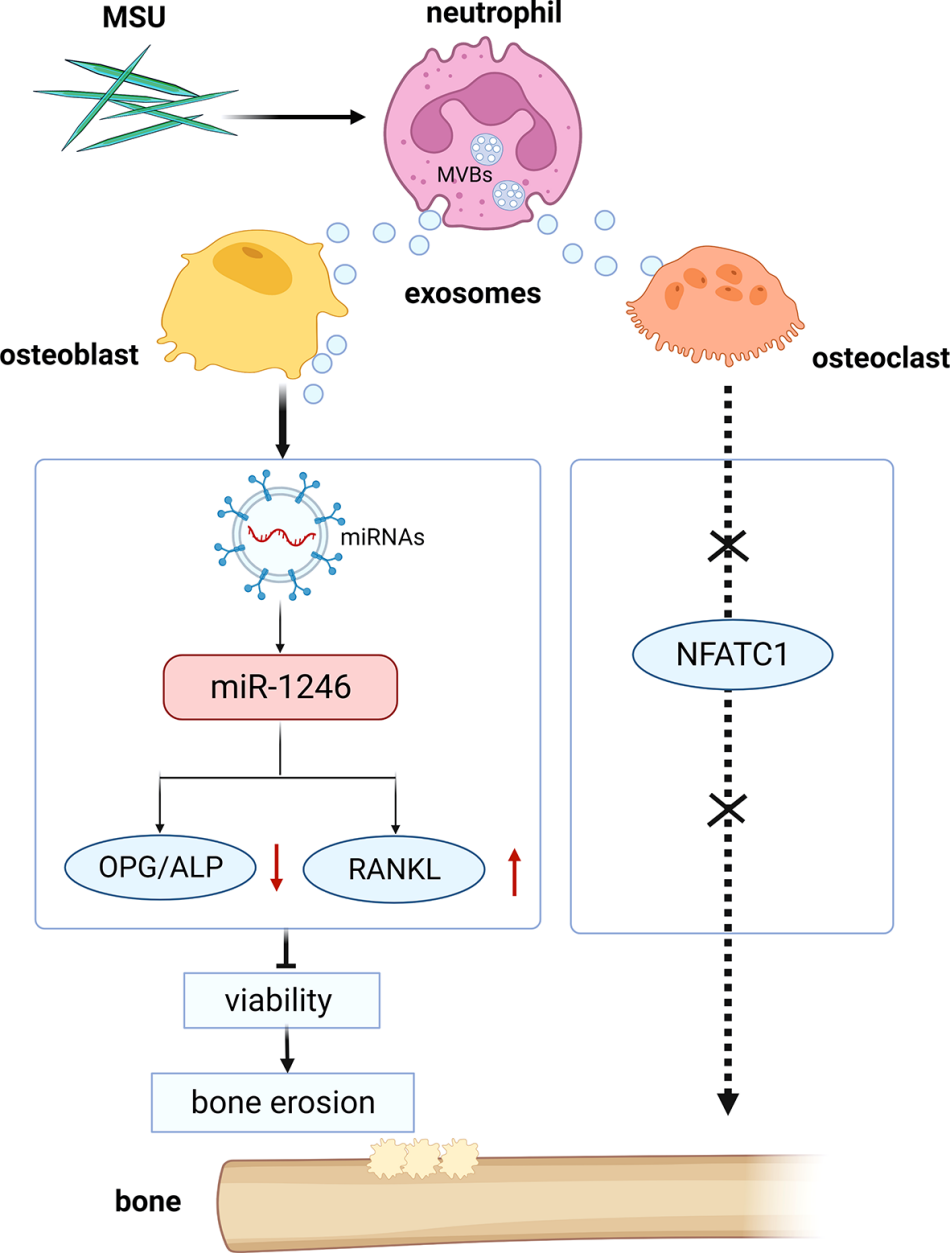
Schematic diagram of the study. Neutrophil-derived miRNA-1246-overexpression exosomes induced by MSU inhibit the viability of osteoblasts.

Exosome-mediated transfer of mRNAs and miRNAs has been shown to influence recipient cells, such as regulating their protein expressions, suggesting an *in vivo* functional role of exosome-derived mRNA and miRNA. Although neutrophil-derived exosomes can enhance the release of anti-inflammatory mediators to exacerbate inflammatory responses, the effects of the exosomes on bone erosion need to be explored (11, 12). Our study showed that neutrophil-derived exosomes stimulated by MSU could inhibit the viability of osteoblasts *via* miRNA-1246 (**Figure 10**). Besides, miRNAs contributed to the regulation of osteoblasts in bone formation through their effects on the bone morphogenetic protein (BMP) pathway (26). A pivotal role of miR-494 in negatively regulating osteogenic differentiation has been recently reported (27). Moreover, miR-100 was identified as a novel regulator of human osteogenesis through its interaction with the target gene, bone morphogenetic protein type II receptor (BMPR2), in human adipose-derived stem cells (hASCs) (28). Although the viability of the osteoclasts was not affected by neutrophil-derived exosomes, the expression level of RANKL was elevated, promoting the viability of osteoclasts. In this study, we only assessed the effects of neutrophil-derived exosomes *in vitro*.

In summary, we found that neutrophil-derived exosomes stimulated by MSU could inhibit the viability of osteoblasts *via* specific miRNAs.

## Data Availability Statement

The datasets presented in this study can be found in online repositories. The name of the repository and accession number can be found below: National Center for Biotechnology Information (NCBI) BioProject, https://www.ncbi.nlm.nih.gov/bioproject/, PRJNA818782.

## Author Contributions

EJ and JZ conceptualized, planned, and designed the study. LZ, XQ, JX, YX, YJ, MX, YZ, JW, and DT carried out the experiments. EJ and HZ drafted and finalized the manuscript. HG assisted in the analysis of data. All authors contributed to the article and approved the submitted version.

## Funding

Shenzhen Science and Technology Plan Project (JCYJ20180302173532311), National Natural Science Foundation of China (82174290), The Sanming Project of Medicine in Shenzhen (SZSM201612080).

## Conflict of Interest

The authors declare that the research was conducted in the absence of any commercial or financial relationships that could be construed as a potential conflict of interest.

## Publisher’s Note

All claims expressed in this article are solely those of the authors and do not necessarily represent those of their affiliated organizations, or those of the publisher, the editors and the reviewers. Any product that may be evaluated in this article, or claim that may be made by its manufacturer, is not guaranteed or endorsed by the publisher.

## Abbreviations

TEM, transmission electron microscopy; NTA, nanoparticle tracking analysis; CCK-8, cell counting kit-8; FCM, flow cytometry; QPCR, quantitative real-time PCR; WB, western blot; ALP, alkaline phosphatase; OPG, osteoprotegerin; RANKL, receptor activator of nuclear factor kappa- B ligand; CFSE, 5(6)-Carboxyfluorescein diacetate succinimidyl ester; miRNAs, microRNAs; MSU, monosodium urate; NFATC1, nuclear factor of activated T cells 1; US, United States; NBF, new bone formation; BME, bone edema; MRI, magnetic resonance imaging; NC, normal contrast; TBST, TBS+Tween; PVDF, polyvinylidene fluoride; GAPDH, Glyceraldehyde-3-phosphate dehydrogenase; BMSCs, bone marrow mesenchymal stem cells; BMPR2, bone morphogenetic protein type II receptor; hASCs, human adipose-derived stem cells.
